# Positive Pivot Shift Test among Patients with Displaced Anterior Tibial Spine Fractures Undergoing Arthroscopic Fixation in a Tertiary Care Centre: A Descriptive Cross-sectional Study

**DOI:** 10.31729/jnma.7995

**Published:** 2023-03-31

**Authors:** Amit Joshi, Sabin Shrestha, Subhash Regmi, Bibek Basukala, Rajiv Sharma, Rohit Bista, Nagmani Singh, Ishor Pradhan

**Affiliations:** 1 Department of Orthopaedics, B&B Hospital Pvt Ltd., Gwarko, Lalitpur, Nepal; 2 Department of Orthopaedics, Nepalgunj Medical College Teaching Hospital, Kohalpur, Banke, Nepal

**Keywords:** *anterior cruciate ligament*, *arthroscopy*, *knee fractures*, *physical examination*

## Abstract

**Introduction:**

Understanding of displaced anterior tibial spine fractures has evolved over the years and is now considered to be anterior cruciate ligament avulsion injuries rather than intra-articular fractures. However, there are very few studies available evaluating the presence or absence of a pivot shift test, which is specific in diagnosing anterior cruciate ligament insufficiency, in patients with anterior tibial spine fractures. This study aimed to find out the prevalence of the positive pivot shift test among patients with displaced anterior tibial spine fractures undergoing arthroscopic fixation in a tertiary care centre.

**Methods:**

A descriptive cross-sectional study was conducted among patients presented with displaced anterior tibial spine fractures undergoing arthroscopic fixation. The data were collected between 1 January 2020 and 30 May 2022. Ethical approval was obtained from the Institutional Review Committee (Reference number: IRC_2019_11_09_1). All patients who presented with displaced anterior tibial spine fractures undergoing arthroscopic fixation were included in the study and those who did not provide consent were excluded. The pivot test was performed under anaesthesia. Point estimate and 90% Confidence Interval were calculated.

**Results:**

Among 48 patients, pivot shift was positive in 36 (75%) (64.75-85.25, 90% Confidence Interval). The mean age of the participants was 28.97±11.16 years, 21 (58.33%) were males and 15 (41.66%) were females.

**Conclusions:**

The prevalence of positive pivot shift test under anaesthesia in patients with displaced anterior tibial spine fractures undergoing arthroscopic fixation was higher than other studies done in similar settings.

## INTRODUCTION

Anterior tibial spine fractures (ATSFs) were thought to be intraarticular fractures.^1^ However, understanding of ATSFs has evolved over the years and is now considered to be anterior cruciate ligament (ACL) avulsion injuries rather than intra articular fractures.^2^ Some researchers suggest that these injuries should be recognized and treated early to avoid ACL insufficiency.^3,4^ However, there is a lack of evidence on either ATSF is equivalent to the avulsion of ACL or not.

It is known from the literature that the pivot shift test has high specificity in diagnosing ACL insufficiency.^5^

This suggests that patients with displaced ATSF should be evaluated with pivot shift tests before any treatment was instituted. However, there are very few studies available evaluating the presence or absence of pivot shift tests in patients with ATSFs.

The aim of this study was to find the prevalence of the positive pivot shift test among patients with displaced ATSF undergoing arthroscopic fixation in a tertiary care centre.

## METHODS

A descriptive cross-sectional study was conducted among patients with displaced ATSFs undergoing arthroscopic fixation admitted to the Department of Orthopaedics, B&B Hospital Private Limited. Ethical approval was obtained from the Institutional Review Committee (IRC) of the same institute (Reference number: IRC_2019_11_09_1). The data were collected between 1 January 2020 and 31 May 2022. Patients with displaced ATSFs undergoing arthroscopic fixation admitted in the Department during the study period were included in the study. Patients who did not provide consent were excluded. Written informed consent was obtained from all the participants and the privacy of the data was kept confidential. A convenience sampling method was used. The sample size was calculated using the following formula:


$$\begin{array}{ll} \text{n} & ={\text{Z}}^{2}\times \frac{\text{p}\times \text{q}}{{\text{e}}^{\text{2}}}\\ & =1.645^{2}\times \frac{0.2\times 0.8}{0.1^{2}}\\ & =44 \end{array}$$

Where,

n = minimum required sample size
Z = 1.64 at 90% Confidence Interval (CI)
p = prevalence of positive pivot shift test among patients with ATSF, 20%^6^
q = 1-p
e = margin of error, 10%

The calculated minimum required sample size was 44. However, a total of 48 patients were included in the study.

All included patients were classified according to Zaricznyj modification of Meyers and Mckeever classification, using plain radiograph and CT scan as type I: minimally/nondisplaced fragment, type II: anterior elevation of the fragment, type III: complete separation of the fragment, type IV: comminuted avulsion or rotation of the fracture fragment.^7^ MRI scan was also obtained prior to evaluation under anaesthesia.

All included patients underwent evaluation under anaesthesia (either spinal anaesthesia or general anaesthesia). After adequate anaesthesia, Lachman and pivot shift test was performed by two fellowship-trained surgeons independently who were blinded to others' findings about the same test. Any disagreement was resolved by a senior arthroscopic and sports surgeon. All the tests were done first in the normal knee and then compared with the affected knee. A soft anterior endpoint with the increased anterior excursion of the tibia was considered positive Lachman test and a hard endpoint was considered negative.^8^ Similarly, a positive pivot shift test was indicated, if there was an abnormal movement of tibial tuberosity because of the reduction of the tibia from a subluxated position at 30-40° of flexion.^9^ All patients underwent diagnostic arthroscopy to verify any associated injury reported in MRI. Appropriate treatment of associated injuries was provided (if any), and suture bridge fixation of ATSF was done.^10^

Demographic data with age, sex, mode of injury, duration of injury, types of ATSFs, concomitant injuries, pivot shift and Lachman test findings, were recorded in the patient's individualised proforma. MRI films were also reviewed by fellowship-trained arthroscopy surgeons to verify reports provided by musculoskeletal radiologists.

Statistical analyses were performed using IBM SPSS Statistics version 16.0. Point estimate and 90% CI were calculated.

## RESULTS

Among 48 patients, the pivot shift test was positive in 36 (75%) cases (64.75-85.25, 90% CI). The mean age of the participants was 28.97±11.16 years, 21 (58.33%) were males and 15 (41.66%) were females. A total of 29 (80.55%) patients have road traffic accidents and 23 (63.88%) have type II injuries (Table 1).

**Table 1:** Baseline characteristics of patients with positive pivot shift (n= 36).

Parameters	Outcomes n (%)
**Age group (years)**	
<15	4 (11.11)
15-30	17 (47.22)
31-45	12 (33.33)
>45	3 (8.33)
**Mode of injury**	
Road traffic accidents	29 (80.55)
2-wheeler	25 (69.44)
4-wheeler	4 (11.11)
Fall	7 (19.44)
**Presentation delay**	
Less than 1 week	12 (33.33)
1 week - 1 month	17 (47.22)
1-3 months	5 (13.88)
More than 3 months	2 (5.55)
**Type of injury**	
Type II	-
Type III	23 (63.88)
Type IV	13 (36.11)
**Side of injury**		
Right	23	(63.88)
Left	13	(36.11)
Concomitant injuries	17	(47.22)
Positive Lachman test	36	(100)

## DISCUSSION

This study found that the pivot shift test was positive in 75% of the cases with displaced ATSFs. The findings observed in this study were higher compared to other international studies, which was around 20-60%.^6,11^ A study conducted in Australia involving 97 patients with ATSFs observed a positive pivot shift test only in 20% of the cases.^6^ Another study conducted in Italy involving 10 patients with displaced ATSFs observed a positive pivot shift test in 60% of the cases.^11^ The higher positivity rate in this study could be due to the exclusion of non-displaced ATSFs, as both previous studies have included all types of ATSFs.^6,11^

Two studies conducted involving adults with displaced ATSFs undergoing arthroscopic fixation reported pivot shift test results during final follow-up evaluations.^12,13^ Although preoperative evaluation using pivot shift test findings were not reported, a study conducted in Egypt involving 28 patients (age range, 16 to 42 years) with displaced ATSFs who underwent arthroscopic reduction and internal fixation by cerclage wire loop observed negative pivot shift test in 96.42% cases at a mean follow-up of 24.1 months.^12^ Similarly, a study conducted in France involving 13 patients (age range, 16-51) with displaced ATSFs who underwent arthroscopic bone suture fixation observed a negative pivot shift test in 100% of cases at a mean follow-up of 41 months.^13^ With these findings, an indirect assumption could be made that all included patients in both studies had positive pivot shift tests pre-operatively. Furthermore, a meta-analysis including 16 studies found that the pivot shift test has the highest specificity (97.5%) and positive likelihood ratio (16.00, 95% CI=7.34-34.87) compared to the Lachman test and anterior drawer test to identify ACL insufficiency.^5^ This suggests that patients with displaced ATSFs have a high likelihood of ACL insufficiency. A metaanalysis also observed that the Lachman test is the most sensitive (87.1%) compared to the pivot shift in diagnosing ACL insufficiency.^5^ In this study, the Lachman test was positive in 100% of patients with positive pivot shift tests. This supports the argument that displaced ATSFs should be considered ACL avulsion injuries.

This study also identified that concomitant injuries were present in 47.22% of the cases with positive pivot shift tests. A study conducted in India involving 26 patients observed concomitant injuries of around 27%.^10^ Similarly, a study conducted in the United Kingdom involving 105 patients observed concomitant injuries of around 25%.^14^ This suggests that the prevalence of concomitant injuries observed in this study was higher compared to other international studies. The reason behind that could be a higher number of patients sustaining injuries due to RTAs in our study compared to falls in previous studies.^10,14^

There are some limitations of this study. Although efforts were made to minimise the risks of bias and examinations were performed by trained clinicians, there is always the possibility of reporting bias, as the procedure was not blinded. In addition, the findings can not be generalised, as some patients with displaced ATSFs who were managed conservatively, or not fit for surgery were excluded. Post-operative or follow-up evaluation of arthroscopic fixation using a suture pull-out technique was not reported which could have strengthened the argument about treating displaced ATSFs as ACL avulsion injuries. However, the study outcomes can certainly help clinicians to change their perspective while managing patients with displaced ATSFs, i.e., considering ATSFs as ACL avulsion injuries rather than just intra-articular fractures.

## CONCLUSIONS

The prevalence of positive pivot shift test under anaesthesia in patients with displaced ATSFs undergoing arthroscopic fixation was higher than that other studies done in similar settings. Although the findings cannot be generalised, clinicians should consider the evaluation of ACL insufficiency in patients with displaced ATSFs.
